# Supplementary material for: A Critical View of the Application of the APEX Software (Aqueous Photochemistry of Environmentally-Occurring Xenobiotics) to Predict Photoreaction Kinetics in Surface Freshwaters
Source: Molecules. 2019 Dec 18;25(1):9. doi: 10.3390/molecules25010009 (PMC7017383; doi:10.3390/molecules25010009)
Supplement: Supplementary file 1 [file molecules-25-00009-s001.zip › 3.2.4_gcc-4.4.0.zip!/3.2.4_gcc-4.4.0/3.2.4_gcc-4.4.0/doc/octave/html/interpreter/Empty-Matrices.html]

Empty Matrices - Untitled


Up: Matrices


---

#### 4.1.1 Empty Matrices

A matrix may have one or both dimensions zero, and operations on empty
matrices are handled as described by Carl de Boor in An Empty
Exercise, SIGNUM, Volume 25, pages 2-6, 1990 and C. N. Nett and W. M.
Haddad, in A System-Theoretic Appropriate Realization of the Empty
Matrix Concept, IEEE Transactions on Automatic Control, Volume 38,
Number 5, May 1993.
Briefly, given a scalar s, an m by
n matrix `M(mxn)`, and an m by n empty matrix
`[](mxn)` (with either one or both dimensions equal to zero), the
following are true:

```
     s * [](mxn) = [](mxn) * s = [](mxn)
     
         [](mxn) + [](mxn) = [](mxn)
     
         [](0xm) *  M(mxn) = [](0xn)
     
          M(mxn) * [](nx0) = [](mx0)
     
         [](mx0) * [](0xn) =  0(mxn)
```

By default, dimensions of the empty matrix are printed along with the
empty matrix symbol, []. The built-in variable
`print_empty_dimensions` controls this behavior.

— Built-in Function: val = **print\_empty\_dimensions** ()  
— Built-in Function: old\_val = **print\_empty\_dimensions** (new\_val)  
> Query or set the internal variable that controls whether the
> dimensions of empty matrices are printed along with the empty matrix
> symbol, []. For example, the expression
>
> ```
>           zeros (3, 0)
> ```
>
> will print
>
> ```
>           ans = [](3x0)
> ```

Empty matrices may also be used in assignment statements as a convenient
way to delete rows or columns of matrices.
See Assignment Expressions.

When Octave parses a matrix expression, it examines the elements of the
list to determine whether they are all constants. If they are, it
replaces the list with a single matrix constant.
